# Supplementary material for: A mixed-methods study to explore the modifiable aspects of treatment burden in Parkinson’s disease and develop recommendations for improvement
Source: PLoS One. 2025 Dec 15;20(12):e0338620. doi: 10.1371/journal.pone.0338620 (PMC12704880; doi:10.1371/journal.pone.0338620)
Supplement: S1 File — (PDF) [file pone.0338620.s001.pdf]

The PD Life Study Survey: Living with Parkinson's

If you would prefer to complete this survey online, go to:
https://www.smartsurvey.co.uk/s/F1L768/

Before starting the survey, please read the participant information sheet included in the study pack. This survey is designed to find out about **your experiences of living with Parkinson's** and other long-term health conditions.

Please answer the questions as they relate to **you and your own health with Parkinson's**. Try to answer each question as best as you can. You may ask for help from your friends or family. You can complete the survey in stages if you wish to. If you are not able to complete the whole survey, answering as many questions as you can would still be helpful.

If you need extra help completing the survey or have any questions, please contact the research team (Dr Qian Tan on 07824 895 791; email q.tan@soton.ac.uk) who will be able to help you.

Where you are required to select an option, please tick the box: ☑

Unless otherwise stated, please only tick one answer for each question.

Once you have completed the survey, **please return it by post using the freepost envelope**. If you would like a summary of the study results, please complete the 'Study Results' information form and return this together with your survey. You can also choose to complete the information form online. Thank you for taking part in this study.

Please tick this box to confirm that you have read the participant information sheet and are consenting to participate in this study: ☐

Section One: About You

1. **How old are you (years)?**

   [ ]

2. **What is your sex?**
   ☐ Male
   ☐ Female
   ☐ Prefer not to say

3. **What is your ethnic group**?
   ☐ White
   ☐ Asian/Asian British
   ☐ Black /African/Caribbean/Black British
   ☐ Mixed/Multiple ethnic groups
   ☐ Other (please specify) ________________________

4. **How would you describe your current marital status?**
   ☐ Single (never married or in a civil partnership)
   ☐ Married or in a civil partnership
   ☐ Divorced or dissolved civil partnership
   ☐ Widowed

5. **How would you describe your current living situation (most of the time)?**
   ☐ I live alone
   ☐ With spouse or partner
   ☐ With another family member(s)
   ☐ With friend(s)
   ☐ Other (please specify) ________________________

6. **What property do you currently live in?**
   ☐ Own property
   ☐ Rented property
   ☐ A relative's home
   ☐ A friend's home
   ☐ Care home (residential or nursing home)

Section One: About You (continued)

7. **How would you best describe the area that you currently live in?**
   ☐ Urban
   ☐ Suburban
   ☐ Rural

8. **What is the first part of your postcode?** ***(please leave the last two letters out; e.g. if your postcode is AA55 1BB then answer as AA55 1)***

   ☐ ☐ ☐ ☐ - ☐ ■ ■

9. **What is your current employment status?**
   ☐ Employed (full or part time)
   ☐ Unemployed
   ☐ Retired
   ☐ Other (please specify) ________________________

10. **Have you given up a full time or part time job because of your Parkinson's?**
    ☐ Yes
    ☐ No

11. **What is your highest education level obtained?**
    ☐ Degree level or above
    ☐ A level or equivalent
    ☐ GSCE level or equivalent
    ☐ No qualification
    ☐ Other qualification (please specify) ________________________

12. **How would you describe your usual access to a car?**
    Please tick the closest description:
    ☐ I have a car that I can drive
    ☐ I can regularly travel (as a passenger or driver) in someone else's car
    ☐ I have little or no access to a car
    ☐ I no longer drive due to my Parkinson's

13. **How would you describe your usual access to technology (smartphone, tablet, laptop and/or computer)?**
    Please tick the closest description:
    ☐ I have access to technology that I can use regularly
    ☐ I have access to technology, but I need help using it
    ☐ I have little or no access to technology

Section Two: Looking After Your Health

We are interested in finding out about the effort you have to make to look after your health and how this impacts on your day-to-day life.

Please tell us how much difficulty you have with the following:

(Please tick the box that most applies to you)

	Extremely Difficult	Very Difficult	Quite Difficult	A little Difficult	Not Difficult	Does not apply
1. Taking lots of medications	☐	☐	☐	☐	☐	☐
2. Remembering how and when to take medication	☐	☐	☐	☐	☐	☐
3. Paying for prescriptions, over the counter medication or equipment	☐	☐	☐	☐	☐	☐
4. Collecting prescription medication	☐	☐	☐	☐	☐	☐
5. Monitoring your medical conditions (e.g. checking your blood pressure or blood sugar, monitoring your symptoms etc.)	☐	☐	☐	☐	☐	☐
6. Arranging appointments with health professionals	☐	☐	☐	☐	☐	☐
7. Seeing lots of different health professionals	☐	☐	☐	☐	☐	☐
8. Attending appointments with health professionals (e.g. getting time off work, arranging transport etc.)	☐	☐	☐	☐	☐	☐
9. Getting health care in the evenings and at weekends	☐	☐	☐	☐	☐	☐
10. Getting help from community services (e.g. physiotherapy, district nurses etc.)	☐	☐	☐	☐	☐	☐
11. Obtaining clear and up-to-date information about your condition	☐	☐	☐	☐	☐	☐
12. Making recommended lifestyle changes (e.g. diet and exercise etc.)	☐	☐	☐	☐	☐	☐
13. Having to rely on help from family and friends	☐	☐	☐	☐	☐	☐

Section Three: Your Parkinson’s and Health

1. **How many years have you had Parkinson’s?**

2. **How does your Parkinson’s affect you?**
☐ No sign of disease
☐ Parkinson’s symptoms on one side of the body
☐ Parkinson’s symptoms on both sides of the body with no balance problems
☐ Mild to moderate Parkinson’s symptoms on both sides of the body with some balance problems but still physically independent
☐ Severe disability but still able to walk or stand unassisted
☐ Wheelchair-bound or bedridden unless assisted

3. **Do you have a named Parkinson’s nurse specialist?**
☐ Yes
☐ No
☐ I am not sure

4. **How easy or difficult is it to get in touch with your Parkinson’s nurse specialist if you had a question or concern about your Parkinson’s?**
☐ Very easy
☐ Easy
☐ Neither easy nor difficult
☐ Difficult
☐ Very difficult
☐ I have not needed to get in touch with my Parkinson’s nurse specialist

5. **Where do you get information about your Parkinson’s?**
(Please tick all that apply)
☐ GP
☐ Parkinson’s specialist doctor
☐ Parkinson’s nurse specialist
☐ Parkinson’s UK website
☐ Parkinson’s UK support group
☐ Online search
☐ Other people with Parkinson’s
☐ Other caregivers of someone with Parkinson’s
☐ I prefer not to search for information
☐ Other (please specify) ________________________

Section Three: Your Parkinson's and Health (continued)

6. **How easy or difficult is it to get information about Parkinson's?**
   ☐ Very easy
   ☐ Easy
   ☐ Neither easy nor difficult
   ☐ Difficult
   ☐ Very difficult

7. **Do you feel you have enough information about Parkinson's?**
   ☐ No, but I *would like* to know more
   ☐ No, but I *choose not* to know more
   ☐ Yes, I have enough information
   ☐ Yes, but I feel I have too much information

8. **How often do you need someone to help you when you read instructions, pamphlets, or other written material from your doctor or pharmacy?**
   ☐ Never
   ☐ Rarely
   ☐ Sometimes
   ☐ Often
   ☐ Always

Section Three: Your Parkinson's and Health (continued)

9. **Other than your Parkinson's, please list down all your other health conditions (e.g. high blood pressure, diabetes, arthritis etc.) if you have any: -**

Section Four: Your Parkinson’s Symptoms

NON-MOVEMENT PROBLEMS IN PARKINSON’S

The movement symptoms of Parkinson’s are well known. However, other problems can sometimes occur as part of the condition or its treatment. It is important that the doctor knows about these, particularly if they are troublesome for you.

A range of problems is listed below. Please tick the box ‘Yes’ if you have experienced it **<u>during the past month</u>**. If you have **<u>not</u>** experienced the problem in the past month tick the ‘No’ box. You should answer ‘No’ even if you have had the problem in the past but not in the past month.

Have you experienced any of the following in the last month?

	Yes	No
**1.** Dribbling of saliva during the day time ……..	☐	☐
**2.** Loss or change in your ability to taste or smell ………………………………………………………	☐	☐
**3.** Difficulty swallowing food or drink or problems with choking …………………………………………..	☐	☐
**4.** Vomiting or feelings of sickness (nausea) …	☐	☐
**5.** Constipation (less than 3 bowel movements a week) or having to strain to pass a stool (faeces) …………………………………………………	☐	☐
**6.** Bowel (faecal) incontinence ……………………	☐	☐
**7.** Feeling that your bowel emptying is incomplete after having been to the toilet …………………	☐	☐
**8.** A sense of urgency to pass urine makes you rush to the toilet …………………………………….	☐	☐
**9.** Getting up regularly at night to pass urine ..	☐	☐
**10.** Unexplained pains (not due to known conditions such as arthritis) …………………………………….	☐	☐
**11.** Unexplained change in weight (not due to change in diet) …………………………………………	☐	☐
**12.** Problems remembering things that have happened recently or forgetting to do things …………….	☐	☐
**13.** Loss of interest in what is happening around you or doing things ………………………………	☐	☐
**14.** Seeing or hearing things that you know or are told are not there ……………………………..	☐	☐
**15.** Difficulty concentrating or staying focussed……………………………………………….	☐	☐

	Yes	No
**16.** Feeling sad, ‘low’ or ‘blue’ ……………………..	☐	☐
**17.** Feeling anxious, frightened or panicky ……	☐	☐
**18.** Feeling less interested in sex or more interested in sex ……………………………………	☐	☐
**19.** Finding it difficult to have sex when you try …………………………………………………	☐	☐
**20.** Feeling lightheaded, dizzy or weak standing from sitting or lying …………………	☐	☐
**21.** Falling …………………………………………………	☐	☐
**22.** Finding it difficult to stay awake during activities such as working, driving or eating ………………………………………………….	☐	☐
**23.** Difficulty getting to sleep at night or staying asleep at night …………………………	☐	☐
**24.** Intense, vivid dreams or frightening dreams ………………………………………………	☐	☐
**25.** Talking or moving about in your sleep as if you are ‘acting’ out a dream ……………	☐	☐
**26.** Unpleasant sensations in your legs at night or while resting, and a feeling that you need to move ………………………………………………	☐	☐
**27.** Swelling of your legs ……………………………	☐	☐
**28.** Excessive sweating ………………………………	☐	☐
**29.** Double vision ……………………………………	☐	☐
**30.** Believing things are happening to you that other people say are not true …………………	☐	☐

Copyright © 2021 International Parkinson and Movement Disorder Society (MDS). All rights reserved. Adapted and used with permission of MDS. This derivative of the NMSQ has not been validated or tested for reliability.

Section Five: Your Medications

1. **Please list down ALL the medications (prescribed and over the counter) that you take for your Parkinson's and/or other health conditions:-**

	Medication Name and Dose	Frequency
1.		
2.		
3.		
4.		
5.		
6.		
7.		
8.		
9.		
10.		
11.		
12.		
13.		
14.		
15.		
16.		
17.		
18.		
19.		
20.		

Section Five: Your Medications (continued)

2. **Does anyone help you with your medications?**
   ☐ Yes
   ☐ No

3. **In total, how many different times a day do you need to take your medications?**

4. **Are you prescribed any medication that requires you to inject yourself?**
   ☐ Yes
   ☐ No

5. **Do you use any of the following to help you remember to take your medications?** ***(Please tick all that apply)***
   ☐ Dosette box/ pill box
   ☐ Medication timers
   ☐ Phone reminders
   ☐ I have someone who helps remind me
   ☐ I do not need reminders
   ☐ Other (please specify) ________________________

6. **How do you manage your prescriptions?**
   ☐ I collect my medications from the pharmacy
   ☐ I have someone who helps me collect medications from the pharmacy
   ☐ My medications are delivered to my home
   ☐ Other (please specify) ________________________

Section Six: Your Use of Healthcare Services

1. ***In the last 12 months*, how many times have you had contact or accessed the following healthcare professionals <u>for your Parkinson's</u>** *(this includes all face-to-face, telephone or video appointments, home visits or other methods)*:

   a) Parkinson's specialist doctor? ☐

   b) Parkinson's nurse specialist? ☐

   c) Physiotherapist? ☐

   d) Occupational therapist? ☐

   e) Speech and language therapist? ☐

   f) Dietician? ☐

   g) Older People Mental Health team? ☐

   h) GP? ☐

2. ***In the last 12 months,* how many times have you had contact or accessed your <u>GP</u> for anything else <u>other than your Parkinson's</u>?** ☐

3. ***In the last 12 months*, how many times have you been to the <u>hospital in an emergency</u>?** ☐

4. ***In the last 12 months*, how many times have <u>paramedics</u> attended your home?** ☐

Section Seven: You and Your Health

1. **Have you felt overstretched by everything you've had to do to manage your health in the last month (e.g. taking medications, getting prescriptions, attending appointments) ?**
   ☐ Yes
   ☐ No

2. **In general, do you have any health problems that require you to limit your activities?**
   ☐ Yes
   ☐ No

3. **In general, do you have any health problems that require you to stay at home?**
   ☐ Yes
   ☐ No

4. **Do you regularly use a stick, walker, or wheelchair to get about?**
   ☐ Yes
   ☐ No

5. **If you need help, can you count on someone close to you?**
   ☐ Yes
   ☐ No

6. **Do you need someone to help you on a regular basis?**
   ☐ Yes
   ☐ No

7. **Who is the main person who helps or supports you on a regular basis?**
   ☐ Spouse/Partner
   ☐ Family member
   ☐ Friend(s)
   ☐ I do not have/need anybody to help me
   ☐ Other (please specify) ________________________

8. **Do you have a paid carer(s) that help with your personal care?**
   ☐ Yes
   ☐ No

Your Health and Well-Being

This survey asks for your views about your health. This information will help keep track of how you feel and how well you are able to do your usual activities. ***Thank you for completing this survey!***

For each of the following questions, please tick the one box that best describes your answer.

1. In general, would you say your health is:

Excellent	Very good	Good	Fair	Poor
☐ 1	☐ 2	☐ 3	☐ 4	☐ 5

2. The following questions are about activities you might do during a typical day. Does <u>your health now limit you</u> in these activities? If so, how much?

		Yes, limited a lot	Yes, limited a little	No, not limited at all
a	<u>Moderate activities</u>, such as moving a table, pushing a vacuum cleaner, bowling, or playing golf………..	☐ 1	☐ 2	☐ 3
b	Climbing <u>several</u> flights of stairs…………….....…..	☐ 1	☐ 2	☐ 3

SF-12v2™ Health Survey © 1992-2002 by Health Assessment Lab, Medical Outcomes Trust and QualityMetric Incorporated. All rights reserved.
SF-12® is a registered trademark of Medical Outcomes Trust.
(IQOLA SF-12v2 Standard, English (United Kingdom) 8/02)

Section Eight: Your Health and Well-Being (continued)

3. During the <u>past 4 weeks</u>, how much of the time have you had any of the following problems with your work or other regular daily activities <u>as a result of your physical health</u>?

	All of the time	Most of the time	Some of the time	A little of the time	None of the time
a Accomplished less than you would like	☐ 1	☐ 2	☐ 3	☐ 4	☐ 5
b Were limited in the kind of work or other activities	☐ 1	☐ 2	☐ 3	☐ 4	☐ 5

4. During the <u>past 4 weeks</u>, how much of the time have you had any of the following problems with your work or other regular daily activities <u>as a result of any emotional problems</u> (such as feeling depressed or anxious)?

	All of the time	Most of the time	Some of the time	A little of the time	None of the time
a Accomplished less than you would like	☐ 1	☐ 2	☐ 3	☐ 4	☐ 5
b Did work or other activities less carefully than usual	☐ 1	☐ 2	☐ 3	☐ 4	☐ 5

5. During the <u>past 4 weeks</u>, how much did pain interfere with your normal work (including both work outside the home and housework)?

Not at all	A little bit	Moderately	Quite a bit	Extremely
☐ 1	☐ 2	☐ 3	☐ 4	☐ 5

6. These questions are about how you feel and how things have been with you <u>during the past 4 weeks</u>. For each question, please give the one answer that comes closest to the way you have been feeling. How much of the time during the <u>past 4 weeks</u>…

	All of the time	Most of the time	Some of the time	A little of the time	None of the time
a Have you felt calm and peaceful?	☐ 1	☐ 2	☐ 3	☐ 4	☐ 5
b Did you have a lot of energy?	☐ 1	☐ 2	☐ 3	☐ 4	☐ 5
c Have you felt downhearted and low?	☐ 1	☐ 2	☐ 3	☐ 4	☐ 5

7. During the <u>past 4 weeks</u>, how much of the time has your <u>physical health or emotional problems</u> interfered with your social activities (like visiting with friends, relatives, etc.)?

All of the time	Most of the time	Some of the time	A little of the time	None of the time
☐ 1	☐ 2	☐ 3	☐ 4	☐ 5

Thank you for completing these questions!

End of Survey

Thank you for completing this survey.

If you would like a summary of the study results, please complete the 'Study Results' form in your study pack and return the form together with the survey using the freepost envelope or complete the form online at: tinyurl.com/PDLifeResults

Please return the survey by post using the freepost envelope available in your study pack.

The PD Life Study Survey: Caring for Someone with Parkinson's

If you would prefer to complete this survey online, go to:
https://www.smartsurvey.co.uk/s/FJOMVS/

Before starting the survey, please read the participant information sheet included in the study pack. This survey is designed to find out about **your experiences of providing care, assistance, or support for someone with Parkinson's**.

Please answer the questions as they relate to **you or the person with Parkinson's you care for** as stated at the start of each section in the survey. Try to answer each question as best as you can. You may ask for help from your friends or family. You can complete the survey in stages if you wish to. If you are not able to complete the whole survey, answering as many questions as you can would still be helpful.

If you need extra help completing the survey or have any questions, please contact the research team (Dr Qian Tan on 07824 895 791; email q.tan@soton.ac.uk) who will be able to help you.

Where you are required to select an option, please tick the box: ☑
Unless otherwise stated, please only tick one answer for each question.

Once you have completed the survey, **please return it by post using the freepost envelope**. If you would like a summary of the study results, please complete the 'Study Results' information form and return this together with your survey. You can also choose to complete the information form online. Thank you for taking part in this study.

Please tick this box to confirm that you have read the participant information sheet and are consenting to participate in this study ☐

Please tick this box to confirm that you help to support or care for someone with Parkinson's ☐

Section One: About You

1. **How old are you (years)?**

2. **What is your sex?**
   ☐ Male
   ☐ Female
   ☐ Prefer not to say

3. **What is your ethnic group?**
   ☐ White
   ☐ Asian/Asian British
   ☐ Black/African/Caribbean/Black British
   ☐ Mixed/Multiple ethnic groups
   ☐ Other (please specify) ________________________

4. **How would you describe your current marital status?**
   ☐ Single (never married or in a civil partnership)
   ☐ Married or in a civil partnership
   ☐ Divorced or dissolved civil partnership
   ☐ Widowed

5. **What is your relationship to the person with Parkinson's that you support and care for on a regular basis?**
   ☐ Spouse/Partner
   ☐ Family member
   ☐ Friend(s)
   ☐ Other (please specify) ________________________

6. **How would you describe your current living situation (most of the time)?**
   ☐ I live alone
   ☐ With spouse or partner
   ☐ With another family member(s)
   ☐ With friend(s)
   ☐ Other (please specify) ________________________

Section One: About You (continued)

7. **What property do you currently live in?**
   ☐ Own property
   ☐ Rented property
   ☐ A relative's home
   ☐ A friend's home
   ☐ Care home (residential or nursing home)

8. **How would you best describe the area that you currently live in?**
   ☐ Urban
   ☐ Suburban
   ☐ Rural

9. **What is the first part of your postcode?** ***(please leave the last two letters out; e.g. if your postcode is AA55 1BB then answer as AA55 1)***

   ☐ ☐ ☐ ☐ - ☐ ■ ■

10. **What is your current employment status?**
    ☐ Employed (full or part time)
    ☐ Unemployed
    ☐ Retired
    ☐ Other (please specify) ________________________

11. **Have you given up a full time or part time job to help support or care for the person with Parkinson's?**
    ☐ Yes
    ☐ No

12. **What is your highest education level obtained?**
    ☐ Degree level or above
    ☐ A level or equivalent
    ☐ GSCE level or equivalent
    ☐ No qualification
    ☐ Other qualification (please specify) ________________________

Section One: About You (continued)

13. **How would you describe your usual access to a car?**
    Please tick the closest description:
    ☐ I have a car that I can drive
    ☐ I can regularly travel (as a passenger or driver) in someone else's car
    ☐ I have little or no access to a car

14. **How would you describe your usual access to technology (smartphone, tablet, laptop and/or computer)?**
    Please tick the closest description:
    ☐ I have access to technology that I can use regularly
    ☐ I have access to technology, but I need help using it
    ☐ I have little or no access to technology

Section Two: Looking After the Health of the Person You Care For

The questions in this section are about ***you*** as someone who looks after someone else with Parkinson's. Please fill in ***your own*** details, not the details of the person with Parkinson's.

We are interested in finding out about the effort **you** have to make to help look after the health of someone with Parkinson's and how this impacts on your day-to-day life.

Please tell us how much difficulty you have with helping the person you care for with the following: (Please tick the box that most applies to you)

	Not difficult	A little difficult	Quite difficult	Very difficult	Extremely difficult	Does not apply
1. Taking lots of medications	☐	☐	☐	☐	☐	☐
2. Remembering how and when they need to take their medication	☐	☐	☐	☐	☐	☐
3. Paying for their prescriptions, over the counter medication or equipment	☐	☐	☐	☐	☐	☐
4. Collecting their prescription medication	☐	☐	☐	☐	☐	☐
5. Monitoring their medical conditions (e.g. checking their blood sugar, monitoring symptoms etc)	☐	☐	☐	☐	☐	☐
6. Arranging their appointments with health professionals	☐	☐	☐	☐	☐	☐
7. Seeing lots of different health professionals	☐	☐	☐	☐	☐	☐
8. Attending appointments with health professionals (e.g. getting time off work, arranging transport etc)	☐	☐	☐	☐	☐	☐
9. Getting health care for them in the evenings and at weekends	☐	☐	☐	☐	☐	☐
10. Getting them help from community services (e.g. physiotherapy, district nurses etc)	☐	☐	☐	☐	☐	☐
11. Obtaining up-to-date information about their medical conditions	☐	☐	☐	☐	☐	☐

	Not Difficult	A Little Difficult	Quite Difficult	Very Difficult	Extremely Difficult	Does Not Apply
12. Making recommended changes to their lifestyle (e.g. diet, exercise etc)	☐	☐	☐	☐	☐	☐
13. Having to rely on help from family and friends	☐	☐	☐	☐	☐	☐
14. Arranging respite care for the person you care for	☐	☐	☐	☐	☐	☐
15. The financial impact of being a carer (e.g. having to give up work, relying on benefits etc)	☐	☐	☐	☐	☐	☐
16. Adjusting your own lifestyle so that you can look after the person you care for	☐	☐	☐	☐	☐	☐

Section Three: You and Your Health

The questions in this section are about ***you*** as someone who looks after someone else with Parkinson's. Please fill in ***your own*** details, not the details of the person with Parkinson's.

1. **Have you felt overstretched by everything you've had to do to help manage their health in the last month (e.g. taking medications, getting prescriptions, attending appointments)?**
   ☐ Yes
   ☐ No

2. **In general, do you have any health problems that require you to limit your activities?**
   ☐ Yes
   ☐ No

3. **In general, do you have any health problems that require you to stay at home?**
   ☐ Yes
   ☐ No

4. **Do you regularly use a stick, walker, or wheelchair to get about?**
   ☐ Yes
   ☐ No

5. **If you need help, can you count on someone close to you?**
   ☐ Yes
   ☐ No

6. **Do you need someone to help you on a regular basis?**
   ☐ Yes
   ☐ No

7. **Do you have a paid carer(s) that helps you with your personal care?**
   ☐ Yes
   ☐ No

8. **Does helping to look after someone with Parkinson's affect your own health?**
   ☐ Yes
   ☐ No

9. **Does your own health affect how you look after someone with Parkinson's?**
   ☐ Yes
   ☐ No

Section Three: You and Your Health (continued)

10. **Please list down all <u>your</u> health conditions (e.g. high blood pressure, diabetes, arthritis etc.) if you have any:-**

Section Four: Caring for Someone with Parkinson's

The questions in this section are about ***you*** as someone who looks after someone else with Parkinson's. Please fill in ***your own*** details, not the details of the person with Parkinson's.

INSTRUCTIONS: The following is a list of statements, which reflect how people sometimes feel when taking care of another person. After each statement, indicate how often you feel that way: never, rarely, sometimes, quite frequently, or nearly always. There are no right or wrong answers.

'Relative' in this section refers to your spouse/partner/family/friend with Parkinson's that you help to care for.

	Never	Rarely	Sometimes	Quite Frequently	Nearly Always
1) Do you feel that because of the time you spend with your relative you don't have enough time for yourself?	☐	☐	☐	☐	☐
2) Do you feel stressed between caring for your relative and trying to meet other responsibilities for your family or work?	☐	☐	☐	☐	☐
3) Do you feel angry towards your relative when you are around him/her?	☐	☐	☐	☐	☐
4) Do you feel that your relative currently affects your relationship with other family members or friends in a negative way?	☐	☐	☐	☐	☐
5) Do you feel strained when you are around your relative?	☐	☐	☐	☐	☐
6) Do you feel your health has suffered because of your involvement with your relative?	☐	☐	☐	☐	☐
7) Do you feel that you don't have as much privacy as you would like because of your relative?	☐	☐	☐	☐	☐
8) Do you feel that your social life has suffered because you are caring for your relative?	☐	☐	☐	☐	☐
9) Do you feel you have lost control of your life since your relative's illness?	☐	☐	☐	☐	☐
10) Do you feel uncertain about what to do about your relative?	☐	☐	☐	☐	☐
11) Do you feel you should be doing more for your relative?	☐	☐	☐	☐	☐
12) Do you feel you could do a better job in caring for your relative?	☐	☐	☐	☐	☐

ZBI © Steven H. Zarit and Judy M. Zarit, 1980-2008. All rights reserved.
ZBI-12 - United Kingdom/English - Version of 28 Nov 08 - Mapi
ID4948 / ZBI-12_AU1.0_eng-GB.doc

Section Five: Obtaining Information About Parkinson's

The questions in this section are about ***you*** as someone who looks after someone else with Parkinson's. Please fill in ***your own*** details, not the details of the person with Parkinson's.

1. **Where do you get information about Parkinson's?**
   (Please tick all that apply)
   ☐ GP
   ☐ Parkinson's specialist doctor
   ☐ Parkinson's nurse specialist
   ☐ Parkinson's UK website
   ☐ Parkinson's UK support group
   ☐ Online search
   ☐ Other people with Parkinson's
   ☐ Other caregivers of someone with Parkinson's
   ☐ I prefer not to search for information
   ☐ Other (please specify) ________________________

2. **How easy or difficult is it to get information about Parkinson's?**
   ☐ Very easy
   ☐ Easy
   ☐ Neither easy nor difficult
   ☐ Difficult
   ☐ Very difficult

3. **Do you feel you have enough information about Parkinson's?**
   ☐ No, but I *would like* to know more
   ☐ No, but I *choose not* to know more
   ☐ Yes, I have enough information
   ☐ Yes, but I feel I have too much information

4. **How often do you need someone to help you when you read instructions, pamphlets, or other written material from your doctor or pharmacy?**
   ☐ Never
   ☐ Rarely
   ☐ Sometimes
   ☐ Often
   ☐ Always

Section Six: About Their Parkinson's and Health

The questions in this section are about ***<u>the person you care for with Parkinson's.</u>*** Please fill in ***<u>their</u>*** details, not your own details.

1. **How many years have they had Parkinson's?**

2. **How does their Parkinson's affect them?**
   ☐ No sign of disease
   ☐ Parkinson's symptoms on one side of the body
   ☐ Parkinson's symptoms on both sides of the body with no balance problems
   ☐ Mild to moderate Parkinson's symptoms on both sides of the body with some balance problems but still physically independent
   ☐ Severe disability but still able to walk or stand unassisted
   ☐ Wheelchair-bound or bedridden unless assisted

3. **Do they have a named Parkinson's nurse specialist?**
   ☐ Yes
   ☐ No
   ☐ I am not sure

4. **How easy or difficult is it to get in touch with their Parkinson's nurse specialist if you had a question or concern about their Parkinson's?**
   ☐ Very easy
   ☐ Easy
   ☐ Neither easy nor difficult
   ☐ Difficult
   ☐ Very difficult
   ☐ I have not needed to get in touch with the Parkinson's nurse specialist

5. **Does the person you care for with Parkinson's also have a paid carer who helps them with personal care?**
   ☐ Yes
   ☐ No

Section Six: About Their Parkinson's and Health (continued)

6. ***In the last 12 months*, have you noticed any problems with their <u>mood</u>?**
   ☐ Yes
   ☐ No

7. ***In the last 12 months*, have you noticed any problems with their <u>memory</u>?**
   ☐ Yes
   ☐ No

8. ***In the last 12 months*, have you noticed if they experienced any <u>hallucinations</u>?**
   ☐ Yes
   ☐ No

9. **Do you support the person with Parkinson's with their medications?**
   ☐ Yes
   ☐ No
   ☐ They do not need help with taking medications

10. **How does the person with Parkinson's manage their prescriptions?**
   ☐ They collect their own medications from the pharmacy
   ☐ I collect their medications from the pharmacy
   ☐ Their medications are delivered to the home
   ☐ Other (please specify) ________________________

Section Six: About Their Parkinson's and Health (continued)

11. **Please list down ALL the medications (prescribed and over the counter) that they take for their Parkinson's and any other health conditions:-**

	Medication Name and Dose	Frequency
**1.**		
**2.**		
**3.**		
**4.**		
**5.**		
**6.**		
**7.**		
**8.**		
**9.**		
**10.**		
**11.**		
**12.**		
**13.**		
**14.**		
**15.**		
**16.**		
**17.**		
**18.**		
**19.**		
**20.**		

Section Six: About Their Parkinson's and Health (continued)

12. **Other than their Parkinson's, please list down all their other health conditions (e.g. high blood pressure, diabetes, arthritis etc.) if they have any: -**

Section Seven: Their Use of Healthcare Services

The questions in this section are about ***the person you care for with Parkinson's.*** Please fill in ***their*** details, not your own details.

1. ***In the last 12 months,* how many times have they had contact or accessed the following healthcare professionals for their Parkinson's** *(this includes all face-to-face, telephone or video appointments, home visits or other methods)*:

   a) Parkinson's specialist doctor? ☐

   b) Parkinson's nurse specialist? ☐

   c) Physiotherapist? ☐

   d) Occupational therapist? ☐

   e) Speech and language therapist? ☐

   f) Dietician? ☐

   g) Older People Mental Health team? ☐

   h) GP? ☐

2. ***In the last 12 months,* how many times have they had contact or accessed their GP for anything else other than their Parkinson's?** ☐

3. ***In the last 12 months*, how many times have they been to the hospital in an emergency?** ☐

4. ***In the last 12 months*, how many times have paramedics attended their home?** ☐

Your Health and Well-Being

This survey asks for your views about your health. This information will help keep track of how you feel and how well you are able to do your usual activities. *Thank you for completing this survey!*

For each of the following questions, please tick the one box that best describes your answer.

1. **In general, would you say your health is:**

Excellent	Very good	Good	Fair	Poor
☐ 1	☐ 2	☐ 3	☐ 4	☐ 5

2. **The following questions are about activities you might do during a typical day. Does <u>your health now limit you</u> in these activities? If so, how much?**

		Yes, limited a lot	Yes, limited a little	No, not limited at all
a	Moderate activities, such as moving a table, pushing a vacuum cleaner, bowling, or playing golf………...	☐ 1	☐ 2	☐ 3
b	Climbing several flights of stairs……………......…...	☐ 1	☐ 2	☐ 3

SF-12v2™ Health Survey © 1992-2002 by Health Assessment Lab, Medical Outcomes Trust and QualityMetric Incorporated. All rights reserved.
SF-12® is a registered trademark of Medical Outcomes Trust.
(IQOLA SF-12v2 Standard, English (United Kingdom) 8/02)

Section Eight: Your Health and Well-Being (continued)

3. During the past 4 weeks, how much of the time have you had any of the following problems with your work or other regular daily activities as a result of your physical health?

	All of the time	Most of the time	Some of the time	A little of the time	None of the time
a Accomplished less than you would like	☐ 1	☐ 2	☐ 3	☐ 4	☐ 5
b Were limited in the kind of work or other activities	☐ 1	☐ 2	☐ 3	☐ 4	☐ 5

4. During the past 4 weeks, how much of the time have you had any of the following problems with your work or other regular daily activities as a result of any emotional problems (such as feeling depressed or anxious)?

	All of the time	Most of the time	Some of the time	A little of the time	None of the time
a Accomplished less than you would like	☐ 1	☐ 2	☐ 3	☐ 4	☐ 5
b Did work or other activities less carefully than usual	☐ 1	☐ 2	☐ 3	☐ 4	☐ 5

5. During the past 4 weeks, how much did pain interfere with your normal work (including both work outside the home and housework)?

Not at all	A little bit	Moderately	Quite a bit	Extremely
☐ 1	☐ 2	☐ 3	☐ 4	☐ 5

Section Eight: Your Health and Well-Being (continued)

6. These questions are about how you feel and how things have been with you <u>during the past 4 weeks</u>. For each question, please give the one answer that comes closest to the way you have been feeling. How much of the time during the <u>past 4 weeks</u>…

	All of the time	Most of the time	Some of the time	A little of the time	None of the time
a Have you felt calm and peaceful?	☐ 1	☐ 2	☐ 3	☐ 4	☐ 5
b Did you have a lot of energy?	☐ 1	☐ 2	☐ 3	☐ 4	☐ 5
c Have you felt downhearted and low?	☐ 1	☐ 2	☐ 3	☐ 4	☐ 5

7. During the <u>past 4 weeks</u>, how much of the time has your <u>physical health or emotional problems</u> interfered with your social activities (like visiting with friends, relatives, etc.)?

All of the time	Most of the time	Some of the time	A little of the time	None of the time
☐ 1	☐ 2	☐ 3	☐ 4	☐ 5

Thank you for completing these questions!

End of Survey

Thank you for completing this survey.

If you would like a summary of the study results, please complete the ‘Study Results’ form in your study pack and return the form together with the survey using the freepost envelope or complete the form online at: tinyurl.com/PDLifeResults

Please return the survey by post using the freepost envelope available in your study pack.
