# Supplementary material for: A mixed-methods study to explore the modifiable aspects of treatment burden in Parkinson’s disease and develop recommendations for improvement
Source: PLoS One. 2025 Dec 15;20(12):e0338620. doi: 10.1371/journal.pone.0338620 (PMC12704880; doi:10.1371/journal.pone.0338620)
Supplement: S2 File — (PDF) [file pone.0338620.s002.pdf]

<u>FOCUS GROUP GUIDE:</u> Exploring ways to reduce the treatment burden and enhance capacity of people with Parkinson's and their caregivers

Introduction from Researcher

1. Give complete name
2. Introduce self as researcher from Academic Geriatric Medicine at the University of Southampton.
3. Give short explanation about the study and purpose of the interview, check understanding or questions.
4. Discuss confidentiality and confirm consent for audio recording.

- *Thank you for agreeing to participate in this focus group as part of the PD Life Study.*
- *You all have an important role in the care of people with Parkinson's, either as someone who has been diagnosed with Parkinson's, as the family or friend that supports of care for someone with Parkinson's, healthcare professional, volunteer, policy maker or manager.*
- *We will use the term 'caregiver' to refer to the person who helps to support and care for someone with Parkinson's in the discussion today.*
- *The term 'treatment burden' is used to describe the effort and day-to-day tasks required to look after the health of people living with a long-term condition such as Parkinson's. This include taking medication, attending appointments, learning about Parkinson's and lifestyle changes such as diet and exercise. The ability to manage these demands is known as 'capacity'.*
- *The focus group today will discuss some of the key issues that impact the treatment burden and capacity in people with Parkinson's and their caregivers. The issues discussed today were gathered from previous stages of the PD Life Study.*
- *The aim of the focus group today is to develop recommendations of ways that we can improve the treatment burden in Parkinson's.*
- *You were all invited to participate as you have an important role in the care of people with Parkinson's.*

Guidelines/Ground Rules

- *There are no right or wrong answers, only differing points of view.*
- *Please use the 'Raise Hand' button during the discussion.*
- *We ask that you respect each other and listen respectfully even if you disagree with what they are saying. Talk to each other and discuss your views. Your point of view is important. My role as moderator will be to guide the discussion.*
- *We ask that you turn off your phones if possible. If you cannot and you must respond to a call, please put the microphone on mute and re-join us as quickly as you can. If you need a short break to take your medications or need a comfort break, please let me know.*
- *We will be recording the conversation today.*

Introduction

- *Firstly, I would like everyone to introduce themselves. Please could you tell us how you would like to be called and what your role is in the care of Parkinson's.*
- *Before we start our discussion, I will give a briefly summarise the main issues of treatment burden and capacity that people with Parkinson's and their caregivers experience. The issues relate to attending appointments and accessing health professionals, getting satisfactory levels of information related to Parkinson's and managing prescriptions and medications.*
- *We will then discuss ways to improve each issue in turn.*

Discussion

Issues that have been found to impact the treatment burden in Parkinson's:

1. **Attending healthcare appointments and interactions with healthcare professionals:**
   - Organising and arranging healthcare appointments were reported to be difficult for PwP and caregivers. At times, unexpected changes to their appointments may be stressful to some people.
     - *How can we improve this?*
   - The national guideline for Parkinson's recommends 6-monthly follow-up appointments. However, PwP and caregivers report dissatisfaction with the frequency of follow-up appointments for their Parkinson's. Some preferred more frequent appointments, whilst others did not.
     - *What do you think might help?*
     - *Are patient's preferences considered when arranging follow-up PD appointments? Should it be?*
   - Due to COVID-19, many healthcare appointments were changed to telephone appointments and continue to be conducted. Yet, some PwP and caregivers report that that they preferred appointments in person due to hearing or speech issues, difficulty describing symptoms over the phone and inability to build rapport with healthcare professionals.
     - *How can we improve this?*
   - The lack of care coordination between different healthcare teams such as between GP and PD specialist, or between hospital and GPs was an issue of treatment burden.
     - *What can be done to improve this?*
   - Difficulties with contacting healthcare professionals for help and advice was reported as an issue of treatment burden in Parkinson's.
     - *How could this be better?*

2. **Information provision**
   - Getting the right levels of information at the right time, and understanding the information provided was another issue of treatment burden in Parkinson's.

- *What do you think about the level of information provided regarding Parkinson's?*
- *What could make this better?*

- PwP and caregivers searched for information online themselves. The amount of information that may not be related to their own situation can be distressing.
  - *How could this be better?*

3. Management of Medications and Prescriptions

- Prescription errors between GPs, PD specialists and pharmacist when there was change in medications were reported. This can be difficult for PwP and caregivers to solve.
  - *How can we improve this?*

- Collecting prescriptions from the pharmacist can be difficult for some PwP and caregivers. Not everyone could rely on someone to collect their prescriptions for them, or had access to delivery services.
  - *What do you think might help?*

- Issues related to remembering when to take medications and frequent changes in medication doses or timings appear to be an issue of treatment burden in Parkinson's.
  - *How could this experience be improved?*

Issues that impact people's capacity to manage treatment burden in Parkinson's:

- Caregivers have an important role in supporting someone with Parkinson's manage their health and activities of daily living. Our research reports that 50% of caregivers experience high treatment burden levels.
  - *How can we improve the experiences of caregivers?*

- Are there other things that impact on the ability of PwP and their caregivers to look after their health?

- Can you think of other ways to help PwP and their caregivers manage their overall health with Parkinson's?

Closing

- Is there anything we haven't asked that you think should be mentioned?
- Final thoughts and reflection.
- We ask that you keep this discussion here today confidential. Thank you for your time and participation in the PD Life Study.
- We will send you a summary of the study results if you have agreed to receive this at the end of the study.
