# Supplementary material for: A mixed-methods study to explore the modifiable aspects of treatment burden in Parkinson’s disease and develop recommendations for improvement
Source: PLoS One. 2025 Dec 15;20(12):e0338620. doi: 10.1371/journal.pone.0338620 (PMC12704880; doi:10.1371/journal.pone.0338620)

People with Parkinson's (PwP) who responded 'a little to extremely difficult' on the Multimorbidity Treatment Burden Questionnaire

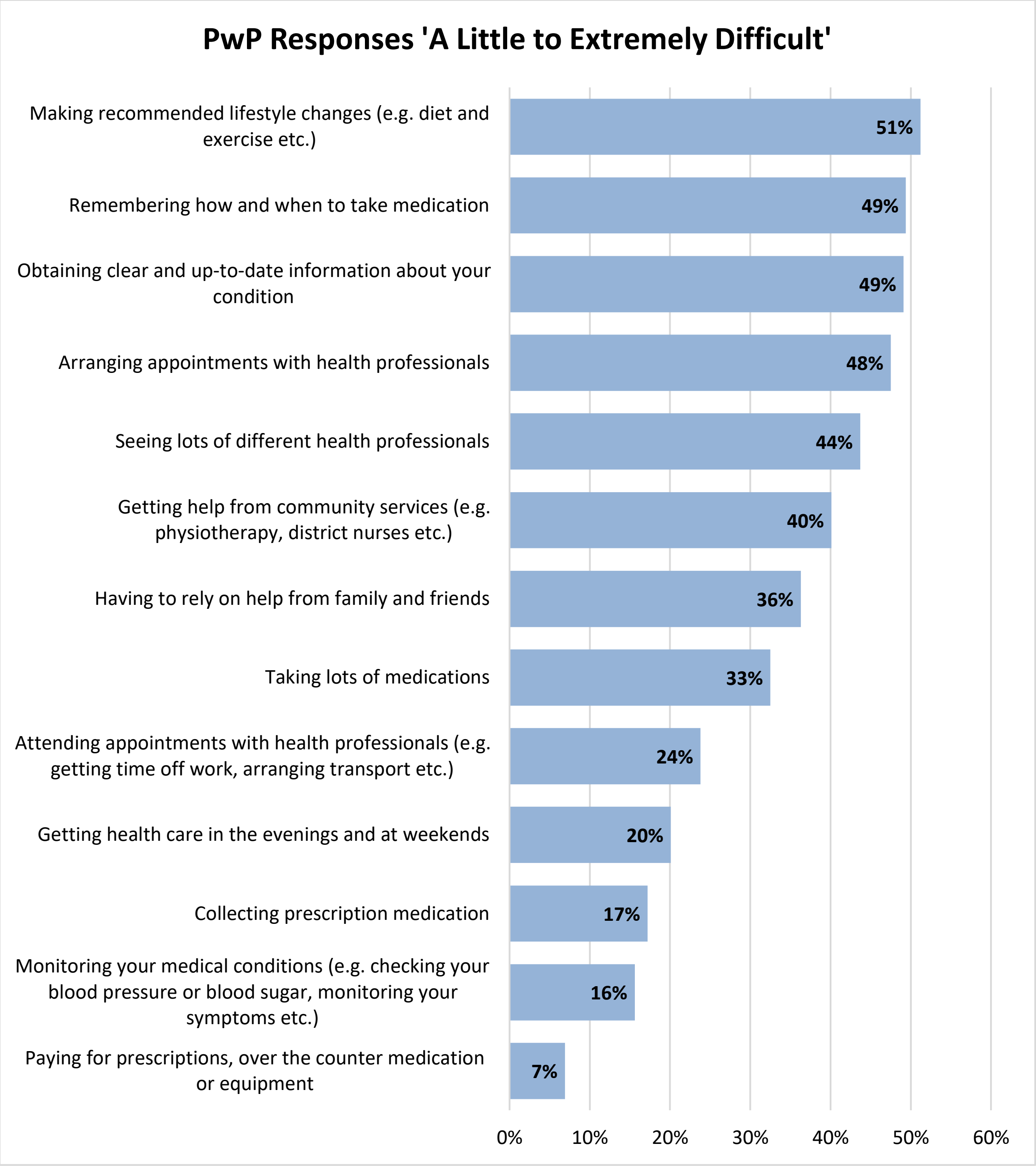

Caregivers who responded ‘a little to extremely difficult’ on the Multimorbidity Treatment Burden Questionnaire

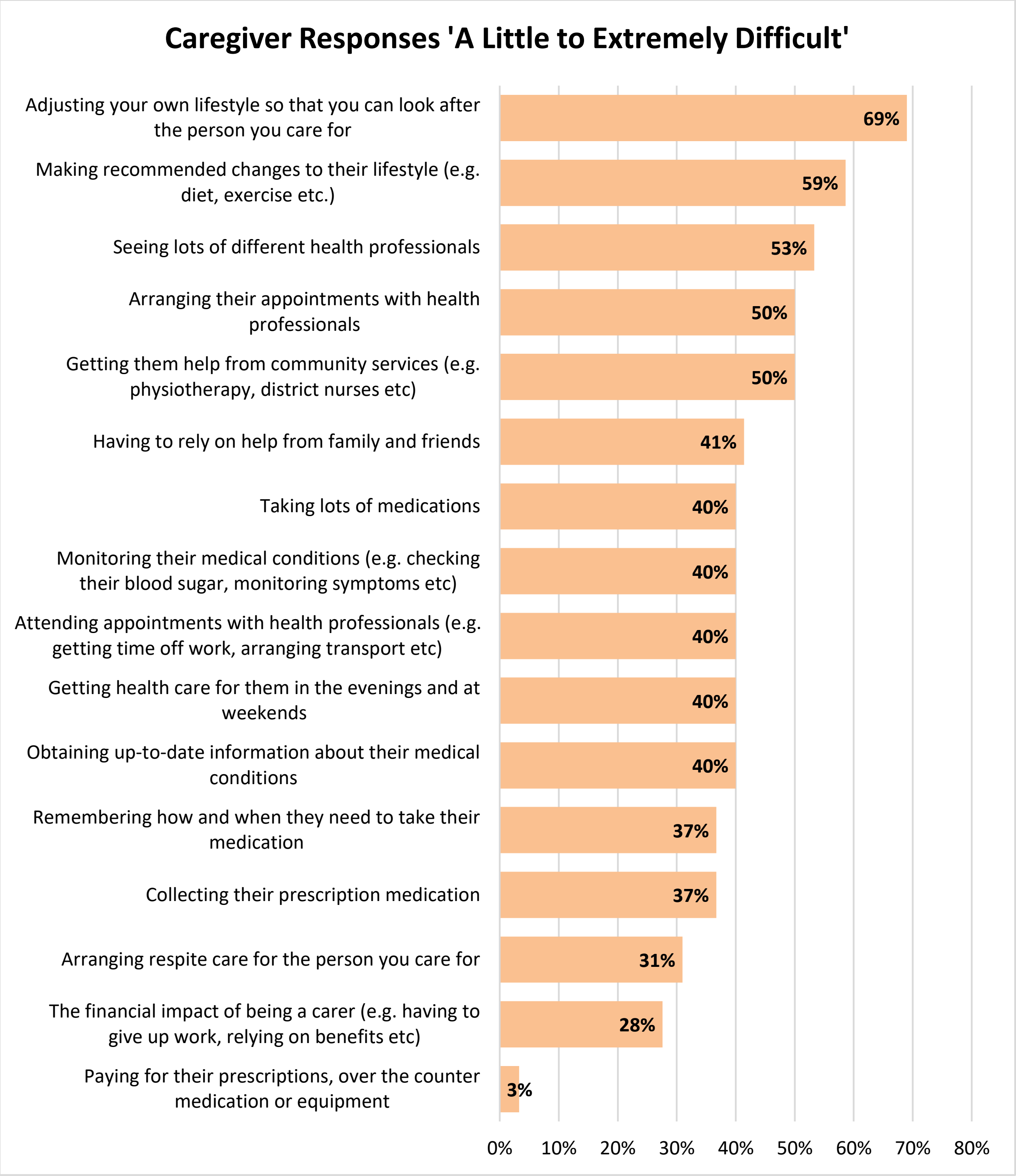

Supplement: S3 File — (PDF) [file pone.0338620.s003.pdf]
