# Supplementary material for: A mixed-methods study to explore the modifiable aspects of treatment burden in Parkinson’s disease and develop recommendations for improvement
Source: PLoS One. 2025 Dec 15;20(12):e0338620. doi: 10.1371/journal.pone.0338620 (PMC12704880; doi:10.1371/journal.pone.0338620)
Supplement: Attachment — Submitted filename: Response to Editor and Reviewers Nov 2025.docx [file pone.0338620.s006.docx]

**Rebuttal letter**

Dear Academic Editor,

Re:

Manuscript Title: *A mixed-methods study to explore the modifiable aspects of treatment burden in Parkinson’s disease and develop recommendations for improvement.*

Thank you for taking your time to review our manuscript and giving us the opportunity to respond to your comments and those of the reviewers. We would also like to thank the reviewers for their constructive feedback which we have worked towards to improve the clarity and quality of the manuscript. The detailed table on the next page depicts how the comments were addressed with corresponding tracked changes highlighted in the attached revised manuscript.

We are confident that our revised manuscript makes a novel contribution to the understanding of treatment burden in Parkinson’s disease, which is an under-researched topic in the literature.

| **No** | **Reviewers’ comments** | **How the comments were addressed** | **Section/Page/Line of where comments were addressed** |
| --- | --- | --- | --- |
| **Journal Requirements/Academic Editor Comments** | | | |
| 1. | *If the reviewer comments include a recommendation to cite specific previously published works, please review and evaluate these publications to determine whether they are relevant and should be cited. There is no requirement to cite these works unless the editor has indicated otherwise.* | We have reviewed the reviewers comments once again and confirm that there have been no recommendations to cite specific previously published works. The additional references (26,26) was added in response to comment No.4 below to justify the rationale of p-value as requested. |  |
| 2. | *Please review your reference list to ensure that it is complete and correct. If you have cited papers that have been retracted, please include the rationale for doing so in the manuscript text, or remove these references and replace them with relevant current references. Any changes to the reference list should be mentioned in the rebuttal letter that accompanies your revised manuscript. If you need to cite a retracted article, indicate the article’s retracted status in the References list and also include a citation and full reference for the retraction notice.* | We have reviewed the reference list and can confirm that no cited papers have been retracted.  We have removed the following reference on review as it was not required in the discussion section: *Eton DT, Ridgeway JL, Linzer M, Boehm DH, Rogers EA, Yost KJ, et al. Healthcare provider relational quality is associated with better self-management and less treatment burden in people with multiple chronic conditions. Patient Prefer Adherence. 2017;11:1635–46. Epub 2017/10/17. doi: 10.2147/ppa.S145942. PubMed PMID: 29033551; PubMed Central PMCID: PMCPMC5630069.* | Discussion Section |
| 3. | *The respected authors have incorporated the reviewers’ edits and comments to the fullest extent possible and have revised the article accordingly. However, it is imperative to emphasize that the manuscript still requires comprehensive linguistic, grammatical, and stylistic editing. The authors are strongly encouraged to undertake a thorough and professional revision to ensure the text adheres to the highest standards of academic writing and clarity.* | Thank you, we have worked hard to incorporate the reviewers’ comments and believe that the manuscript has now been strengthened further. | Whole manuscript |
| **Reviewer #1** | | | |
| 4. | *Clarification of the reasoning behind setting the P-value at 0.25*  *The statistical rationale and significance need to be explained.* | We agree with this comment. We have now included an explanation of the pre-screening stage and justification for the less stringent p-value (p<0.25) for the univariable analysis. This is referenced in the manuscript. | Page 7, Lines 165-169 |
| 5. | *Difference in the accuracy of responses between online and paper surveys. Analysis supporting the reliability and consistency of the data should be provided.* | Thank you for your suggestion. From a total of 192 surveys, there were 31 paper surveys returned. With the small sample of paper surveys, analysis comparing online and paper surveys were not conducted. We have now included this in the manuscript.  As stated in Page 5 Lines 113-114, all paper survey responses were manually double entered into the data spreadsheet to ensure accuracy and reliability. | Page 7, Lines 148-150  Page 5, Lines 113-114 |
| 6. | *Accuracy of PD patients and caregivers in responding to medical information. A detailed explanation of evaluation methods used to assess their understanding and response accuracy is necessary.* | We agree with this comment and recognise this limitation in the manuscript. The use of self-reported data for long-term conditions and variations with medical records was stated in the discussion (Lines 344). We have now expanded this further in the ‘Strengths and Limitation’ section of the paper and included suggestions for future studies. | Page 21, Lines 341-343  Page 28, Lines 478-481 |
| 7. | *MIBQ’s four-category analysis and confirmation of linear relationships with influencing factors. Analysis should elucidate the linear relationships between severity and influencing factors using MIBQ* | Thank you for this comment, the categorical analysis has been determined based on the development of MTBQ questionnaire. . Please see publication: Duncan P, Murphy M, Man M*, et al.* Development and validation of the Multimorbidity Treatment Burden Questionnaire (MTBQ) *BMJ Open* 2020;**8:**e019413. doi: 10.1136/bmjopen-2017-019413  The MTBQ research team recommend the use of four-categories due to skewness of global MTBQ scores, with proportion of patients within each category reported. This has been validated further by the research team in a 2023 publication: Duncan P, Scott LJ, Dawson S*, et al*  Further development and validation of the Multimorbidity Treatment Burden Questionnaire (MTBQ)  *BMJ Open* 2024;**14:**e080096. doi: 10.1136/bmjopen-2023-080096  As such, analysis depicting the linear relationships between severity of treatment burden and variable was not considered to be appropriate for this study. The justification for the dichotomisation of categories for analysis is described in the Methods. | Page 7, Lines 152-158 |
| 8. | *Concerns regarding the small number of PD patients in Phase 2 focus groups and generalizability. Addressing the bias and clarifying the representativeness of these patients are essential.* | Thank you for your comment. Whilst there were three PD patients (out of 12 participants) in the focus groups, the small sample size of PD patients in the focus groups has been added as a limitation in the discussion leading to the lack of generalisability of the study findings. However, focus groups with carers/PD patients alongside healthcare professionals should be seen as a strength given the opportunity to discuss varying views and experiences. | Page 27, Lines 471-473 |
| 9. | *Comparison with similar studies and the novel findings of this study. Clear explanations of the study’s uniqueness, new findings, and their clinical impact are required, along with the perspective of comparison.* | Thank you, we have further emphasised the novel findings and comparisons with previous literature in the discussion section. We have also included a subsection on ‘Implications for Clinical Practice’ in the Discussion. | Discussion section, Page 21  Implications for clinical practice section,  Page 24 |
| 10. | *Simplification and clarification of the text. The text should be refined to be more concise and clear, improving understanding for readers.* | Thank you, we have amended sentences to ensure it is concise and clear where appropriate. | Whole manuscript |
| **Reviewer #2** | | | |
| 11. | *This manuscript addresses an important and timely topic — the treatment burden experienced by people with Parkinson’s disease and their caregivers. The mixed-methods approach is generally appropriate, and the study is clearly presented. However, in its current form, the work does not provide sufficient novelty or depth to justify publication.* | Thank you, we agree that this is an important and timely topic. Treatment burden specific to Parkinson’s and associated factors have not be quantified prior to this study and is therefore a novel finding. Equally the association with frailty and treatment burden has not been identified in previous studies.  Our study highlights that treatment burden in commonly experienced and could therefore lead to poor outcomes in this population. 84% of people with Parkinson’s and 80% of caregivers reported treatment burden. This study highlights key determines of treatment burden, with recommendations for change in clinical practice that can reduce this. We have amended the discussion section to describe specific recommendations for change in clinical practice.  Following this, we believe the updated paper addressing the previous reviewers’ comments has strengthened the paper, highlighting its novelty with clear merits for publication. | Whole manuscript |
| 12. | *The survey results largely confirm known associations (e.g., higher burden with greater disease severity, frailty, and polypharmacy). The second phase of the study — the focus groups — is based on a very small and heterogeneous sample (only 11 participants, including just one caregiver), which limits the robustness of the findings. The output of the qualitative work remains purely narrative, without structured outcome measures or any attempt to assess the practical impact of the recommendations.* | Thank you for this comment. We have amended the discussion section to improve clarity on how the associations found in this study that are specific to treatment burden levels in PD. We have included a subsection that includes implications of our findings that can lead to practical change in clinical practice.  We recognise the limitations the reviewer mentions but, as per our responses to other reviewer comments we have further highlighted the areas of novelty in this under-researched field. Furthermore, treatment burden in patients with long-term conditions remains an under-researched area and this adds valuably to the literature. | Discussion section, Page 21  Implications for clinical practice section,  Page 24 |
| 13. | *Moreover, the proposed suggestions, while sensible, are relatively generic and lack strong grounding in the data. Without any follow-up or validation of these recommendations, the contribution remains modest and speculative. In summary, while the topic is important and the manuscript is well written, the study lacks the scientific relevance and originality required for publication.* | Thank you for your comment. We have made significant changes to the discussion section in response to the reviewer comments above that address these issues. | Discussion section, Page 21 |
